# Supplementary material for: Synaptic vesicle characterization of iPSC-derived dopaminergic neurons provides insight into distinct secretory vesicle pools
Source: NPJ Parkinsons Dis. 2025 Jan 9;11:16. doi: 10.1038/s41531-024-00862-4 (PMC11718109; doi:10.1038/s41531-024-00862-4)
Supplement: Supplementary file 1 — Supplementary Material [file 41531_2024_862_MOESM1_ESM.pdf]

Suplementary Table 1. List of antibodies/dyes used in this study.

Target Protein/dye	Company; Catalog number	Antibody species	Working dilution for immunofluorescence	Working dilution for immunoblotting
βIII-tubulin (TUJ1)	BioLegend; 801201	Mouse	1:500	1:3000
βIII-tubulin	Abcam; ab18207	Rabbit	1:500	N/A
α-Tubulin	Sigma Aldrich; T5168	Mouse	N/A	1:10000
Tyrosine Hydroxylase	EMD Millipore; AB152	Rabbit	1:300	N/A
VAMP2	Synaptic Systems; 104211	Mouse	1:100	N/A
Synapsin I	Synaptic Systems; 106103	Rabbit	1:250<br>1:100 (for EM)	N/A
Synapsin I	Synaptic Systems;10611	Mouse	1:250	N/A
DARPP32	Cell Signaling Technology; 2306S	Rabbit	1:300	1:1000
vGLUT2	Chemicon International; MAB5504	Mouse	1:250	1:1000
VMAT2	Alomone labs; AMT-006	Rabbit	N/A	1:1000
VGAT	Calbiochem; 676780	Guinea Pig	N/A	1:250
PSD95	Sigma Aldrich; 04-1066	Rabbit	1:200	N/A
PSD95	Synaptic Systems;124011	Mouse	1:200	N/A
GM130	BD bioscience; 610822	Mouse	1/1000	N/A
TGN46	Thermo Scientific; PA5-23068	Rabbit	1/1000	N/A
AP2M1	Cell Signaling Technology; 68196T	Rabbit	N/A	1:1000
AP3D1	ABclonal; A13058	Rabbit	N/A	1:1000
Synaptophysin	Synaptic Systems; 101002	Rabbit	1:500<br>1:100 (for EM)	N/A
Synaptophysin	Synaptic Systems; 101001	Mouse	1:250	N/A
Dopamine transporter	EMD Millipore; MAB369	Rat	1:500	1:1000
Rab3	Synaptic Systems; 109111	Mouse	1:250	N/A
Vinculin	Sigma-Aldrich; V4505	Mouse	N/A	1:1000
FLAG	Sigma-Aldrich; F3165	Mouse	1:1000	N/A

Fluo-4	Thermo Fischer Scientific; F14201	N/A	1:1000 (stock: 1 mM)	N/A
HA	Roche; 2131900	Rat	1:50 (for EM)	1:1000
Protein A Gold	UMC Utrecht; PAG 5 nm	Rabbit	1:10 (for EM)	N/A
Collidal Gold AffiniPure Goat Anti-Rat IgG (H+L)	Jackson ImmunoResearch ; 6nm, 112-195-167	Goat	1:10 (for EM)	N/A

Supplementary Figure 1

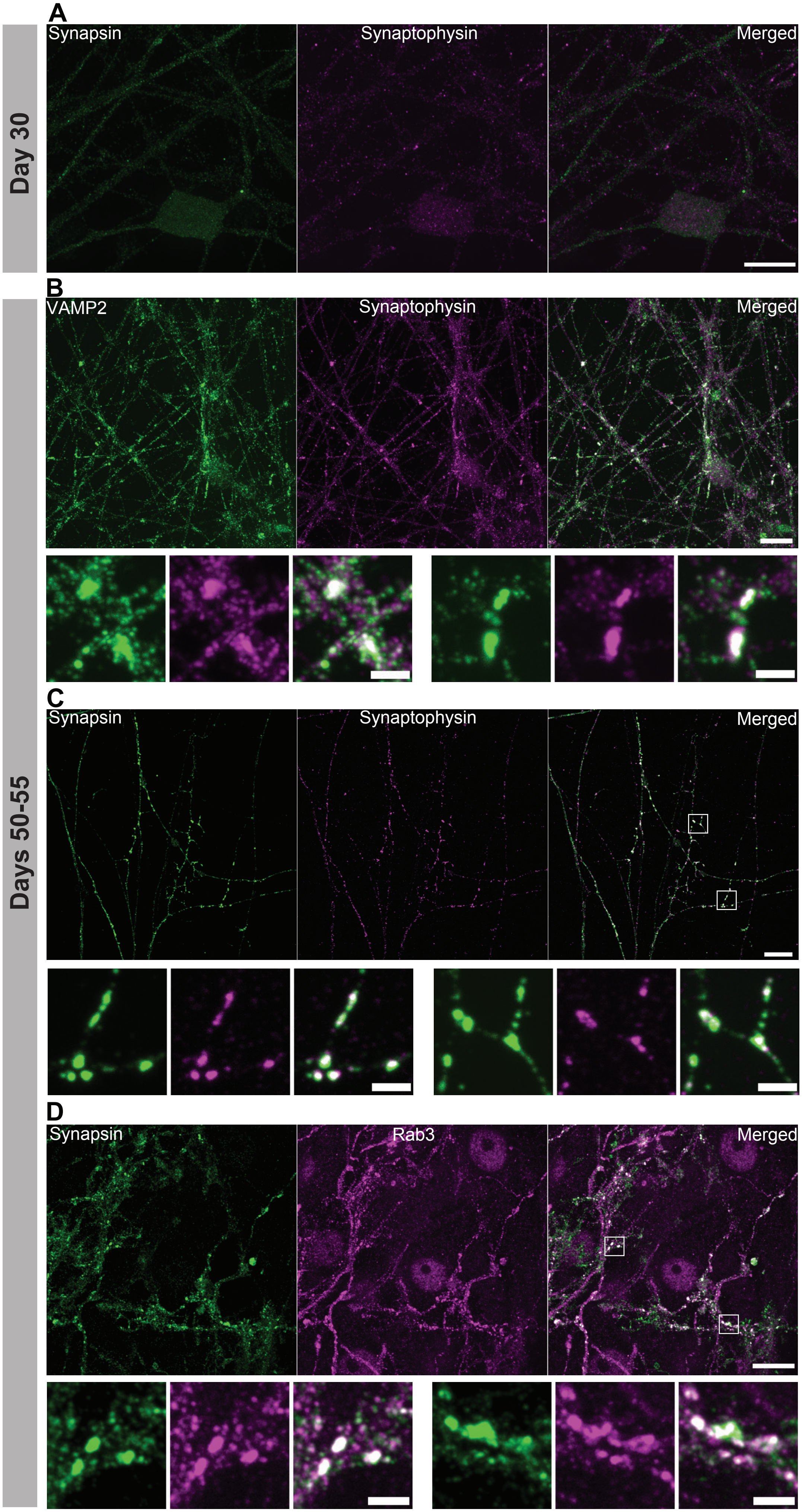

Supplementary Figure 1: Colocalization of other synaptic markers in DA neurons

(A) Representative fluorescence image of DA neurons (day 30) immunolabeled with antibodies against synapsin (green) and synaptophysin (magenta). (B-D) At day 50-55, DA neurons were immunolabeled with antibodies directed to the following synaptic protein combinations: (B) VAMP2 (green) and synaptophysin (magenta), (C) synapsin (green) and synaptophysin (magenta) or (D) synapsin (green) and Rab3 (magenta). Scale bars, 10 μm. Two high magnification images are shown below each panel showing overlapping fluorescence intensities (white) for the aforementioned synaptic markers. Scale bars, 2 μm.

Supplementary Figure 2

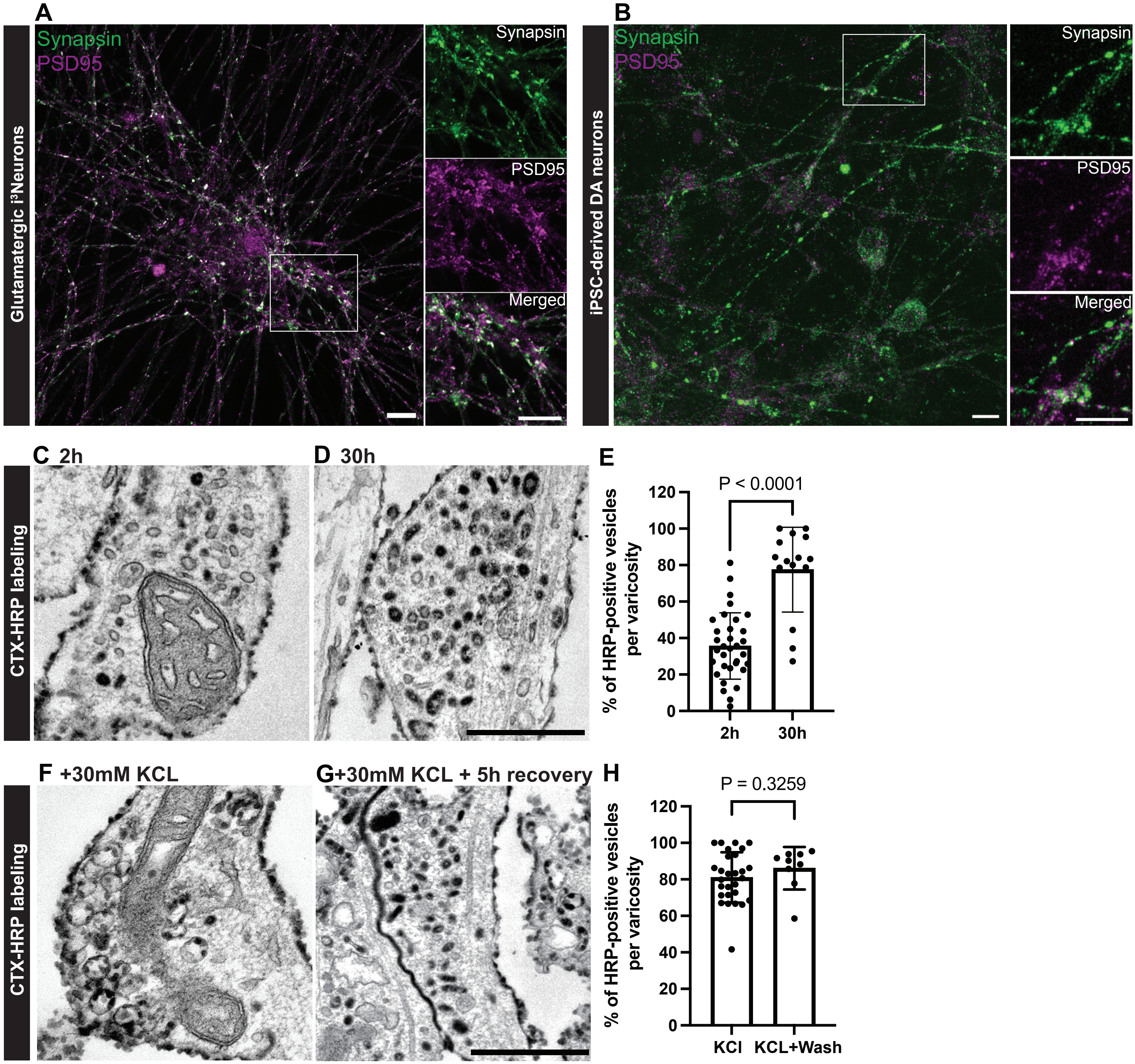

Supplementary Figure 2: Additional characterizations of classical synapses and synaptic boutons in i^{3}Neurons and DA neurons.

(A and B) Representative fluorescence image of i^{3}Neurons (A, day 19) and DA neurons (B, day 50) immunolabeled with antibodies against synapsin (green) and PSD95 (magenta). Enlarged images from boxed areas are shown on the right of each neuronal type, respectively. Scale bars, 10 μm. (C and D) EM images of DA neurons (day 50-55) incubated with CTX-HRP for 2 hours (C) and 30 hours (D) show dark staining of both small and large vesicles. Dark stainings indicate HRP-reactive labeling of vesicles originating from the plasma membrane. Scale bar, 500 nm. (E) Percentage of HRP-positive vesicles per varicosity represented as mean ± SD pooled from ≥448 vesicles in bouton-like structures. (F and G) High K+ stimulation induced stimulus-dependent bulk endocytosis, as a result, most of these organelles were positive for HRP (F). During the recovery period (5 hours), bulk endosomes are converted to SSVs and large vesicles that were positive for HRP (G). Scale bar, 500 nm. (H) Percentage of HRP-positive vesicles per varicosity represented as mean ± SD pooled from ≥400 vesicles in bouton-like structures.

Supplementary Figure 3

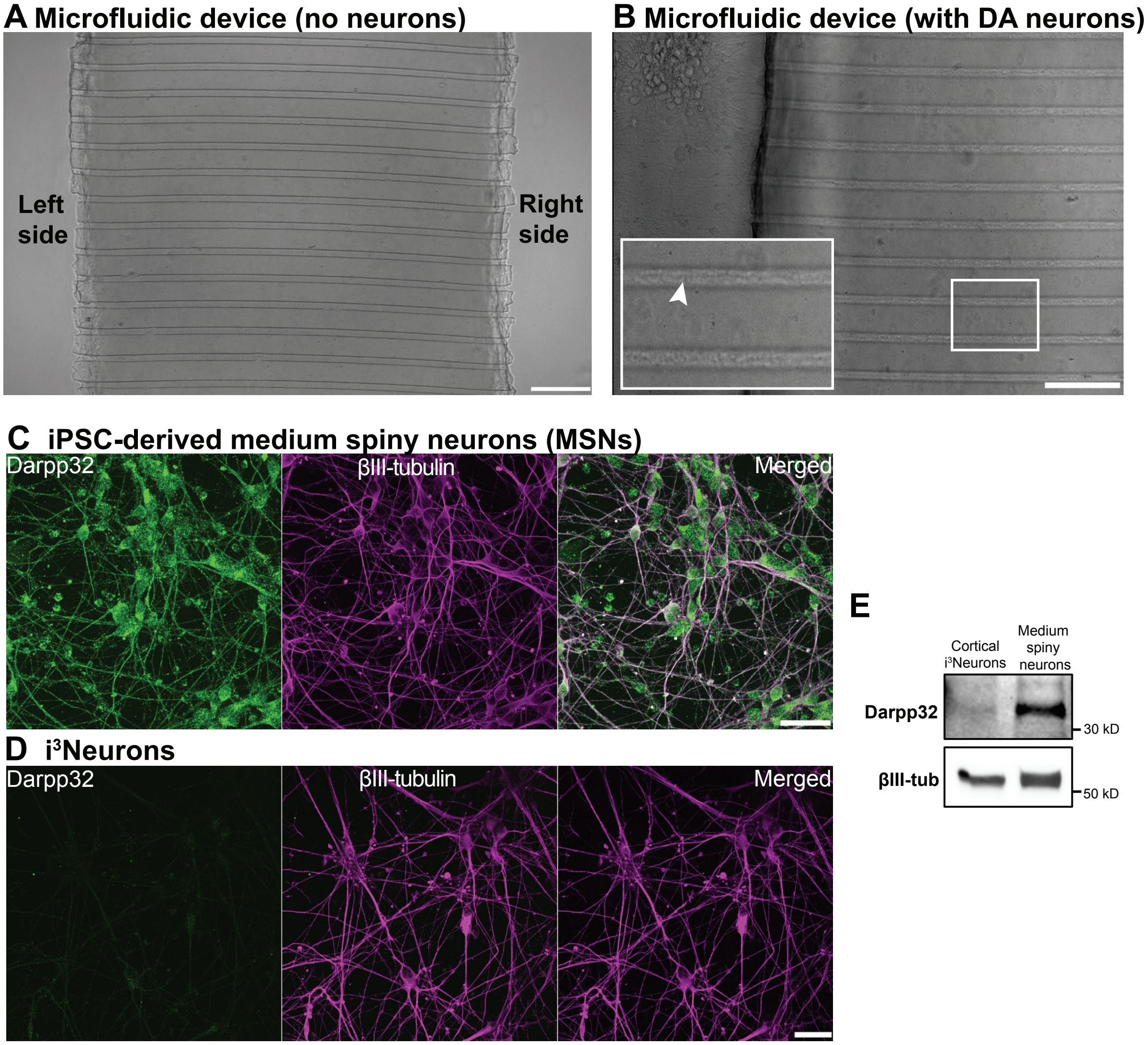

Supplementary Figure 3: Co-culture microfluidic compartmentalization device set-up

(A and B) A compartmentalization device with two chambers (left and right sides) interconnected by a narrow microfluidic channel visualized before (A) and after seeding of DA neurons on one side of the chamber (B). Inset shows arrows (white) indicating axonal outgrowth in the microchannels. Scale bars, 100 μm. (C and D) iPSC-derived MSNs (day 20, C) from Brainxell and i^3Neurons (day 19, D) were immunolabeled with antibodies directed against darpp32 (green) and βIII-tubulin (magenta). Note the strong immunoreactivity of darpp32 in MSNs but not in i^3Neurons. Scale bars, 10 μm. (E) Anti-darpp32 and βIII-tubulin (loading control) western blot of i^3Neurons and MSNs (20 days in cultures).

Supplementary Figure 4

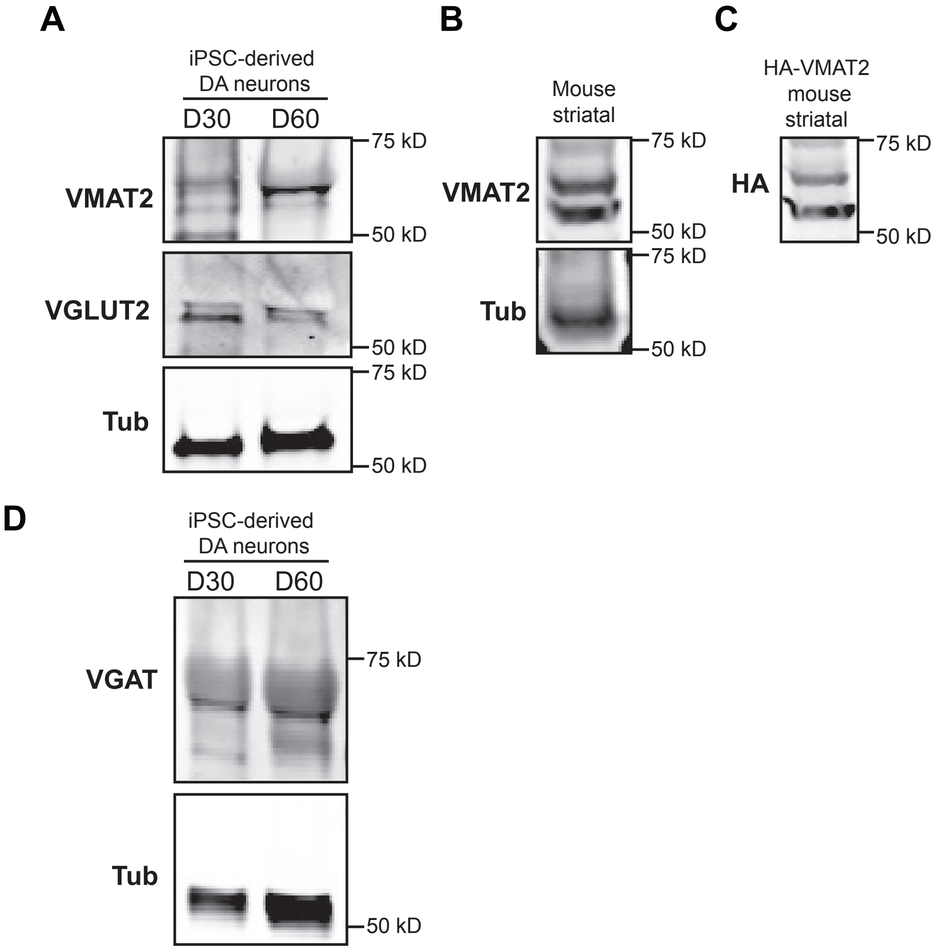

Supplementary Figure 4: iPSC-derived DA neurons express VMAT2, VGLUT2 and VGAT.

(A) Anti-VMAT2, anti-VGLUT2, and anti-βIII-tubulin (loading control) western blot of iPSC-derived DA neurons at day 30 and 60. (B) Western blot of Anti-VMAT2 and anti-βIII-tubulin (loading control) of wildtype mouse striatal lysates. (C) Anti-HA western blot of HA-VMAT2 transgenic mouse striatal lysates. (D) Anti-VGAT and anti-βIII-tubulin (loading control) western blot of iPSC-derived DA neurons at day 30 and 60. Note that mice showed a double band staining for striatal VMAT2, while iPSC-derived DA neurons showed a single band. Region-depended VMAT2 expression has been previously documented (see results section).

Supplementary Figure 5

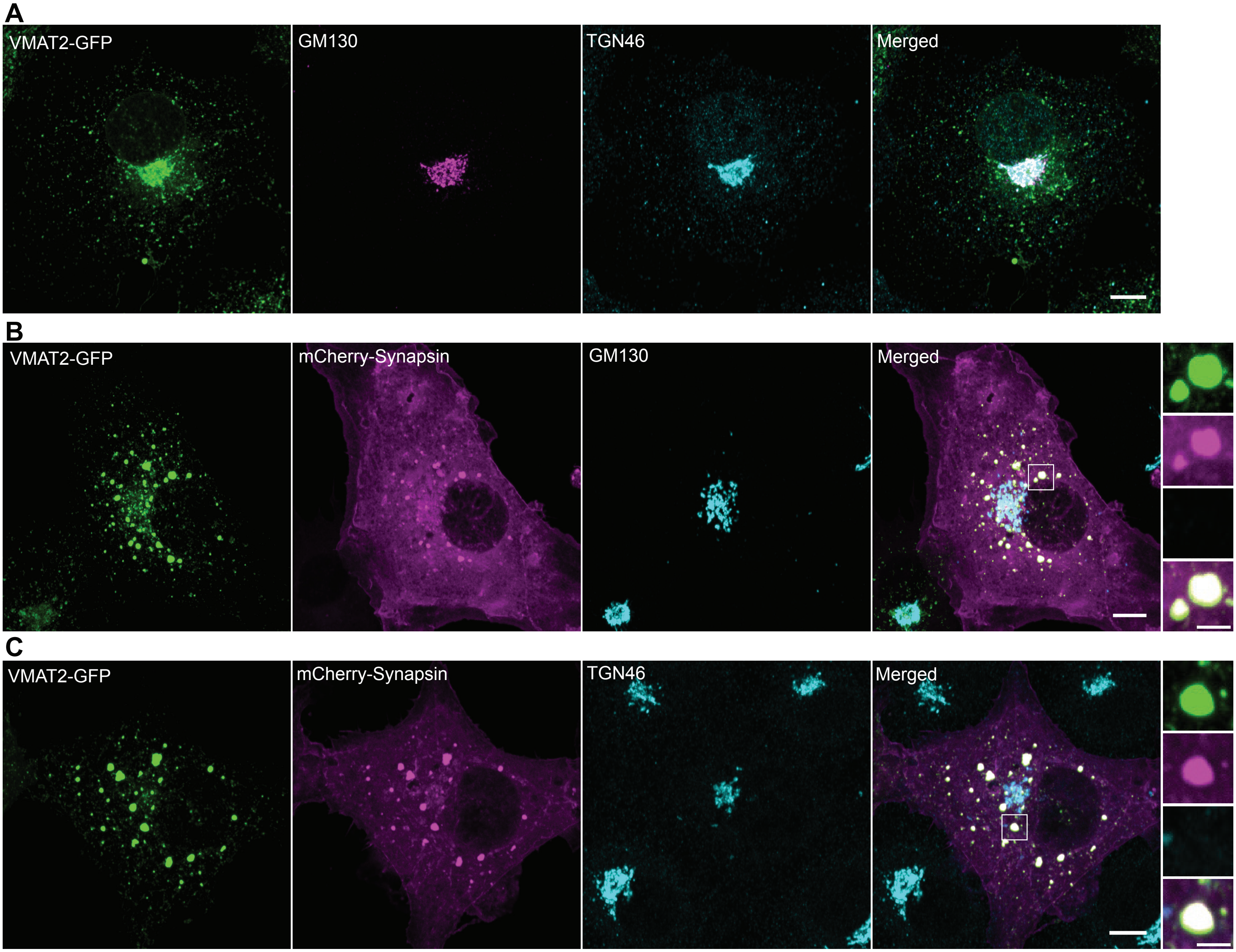

Supplementary Figure 5: Tagged-VMAT2 localizes to the Golgi complex

(A) Representative fluorescence images of COS7 cells transfected with VMAT2-GFP (green), immunolabelled for the *cis*-golgi marker GM130 (magenta), and the *trans*-Golgi marker TGN46 (cyan) antibodies. VMAT2 fluorescence signals overlap with both Golgi markers. (B and C) COS7 cells transfected with VMAT2-GFP (green) and mCherry-synapsin (magenta) and immunolabelled for either GM130 (B, cyan) or TGN46 (C, cyan). The VMAT2-synapsin condensates did not colocalize with the Golgi markers. Scale bars, 10 μm; inset 2.5 μm.

Supplementary Figure 6

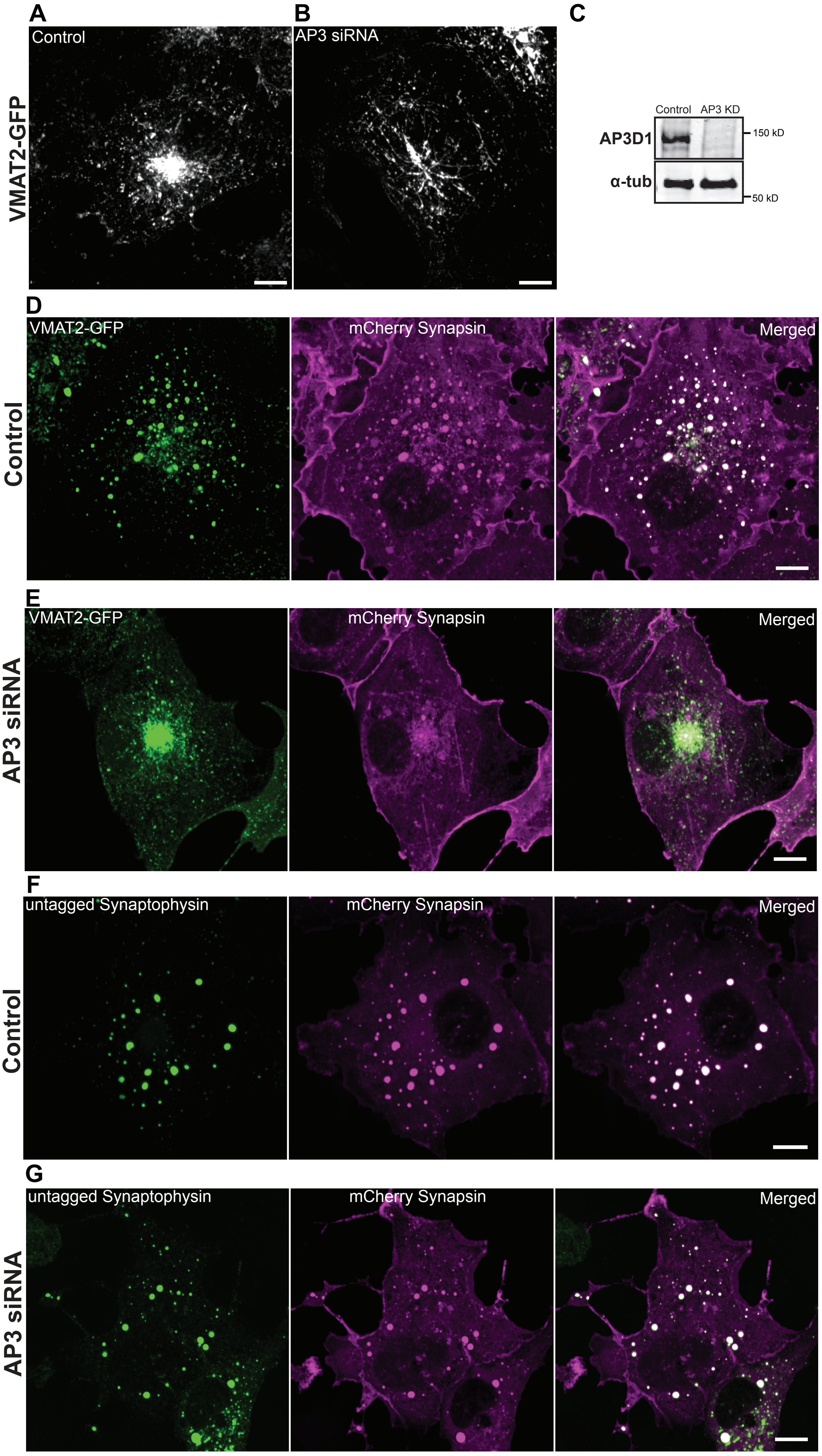

Supplementary Figure 6: AP3 knockdown abolished VMAT2-synapsin but not synaptophysin-synapsin vesicle clusters

(A and B) Representative fluorescence images of COS7 cells transfected with VMAT2-GFP and either control siRNA (A) or AP3D1 siRNA (B) visualized 48 hours post-transfection. Scale bars, 10 μm. (C) Anti-AP3D1 and α-tubulin (loading control) western blot of control and AP3 KD siRNA-treated COS7 cells 48 hours post-transfection. (D and E) COS7 cells transfected with VMAT2-GFP (green) and mCherry-synapsin (magenta) treated with control siRNA (D) or AP3D1 siRNA (E). (F and G) COS7 cells transfected with untagged synaptophysin (green) and mCherry-synapsin (magenta) treated with control siRNA (F) or AP3D1 siRNA (G). Untagged synaptophysin was revealed by immunofluorescence. Images from D-G were taken 48 hours after transfection. Note the lack of VMAT2-synapsin droplets in E. Scale bars, 10 μm.

Supplementary Figure 7

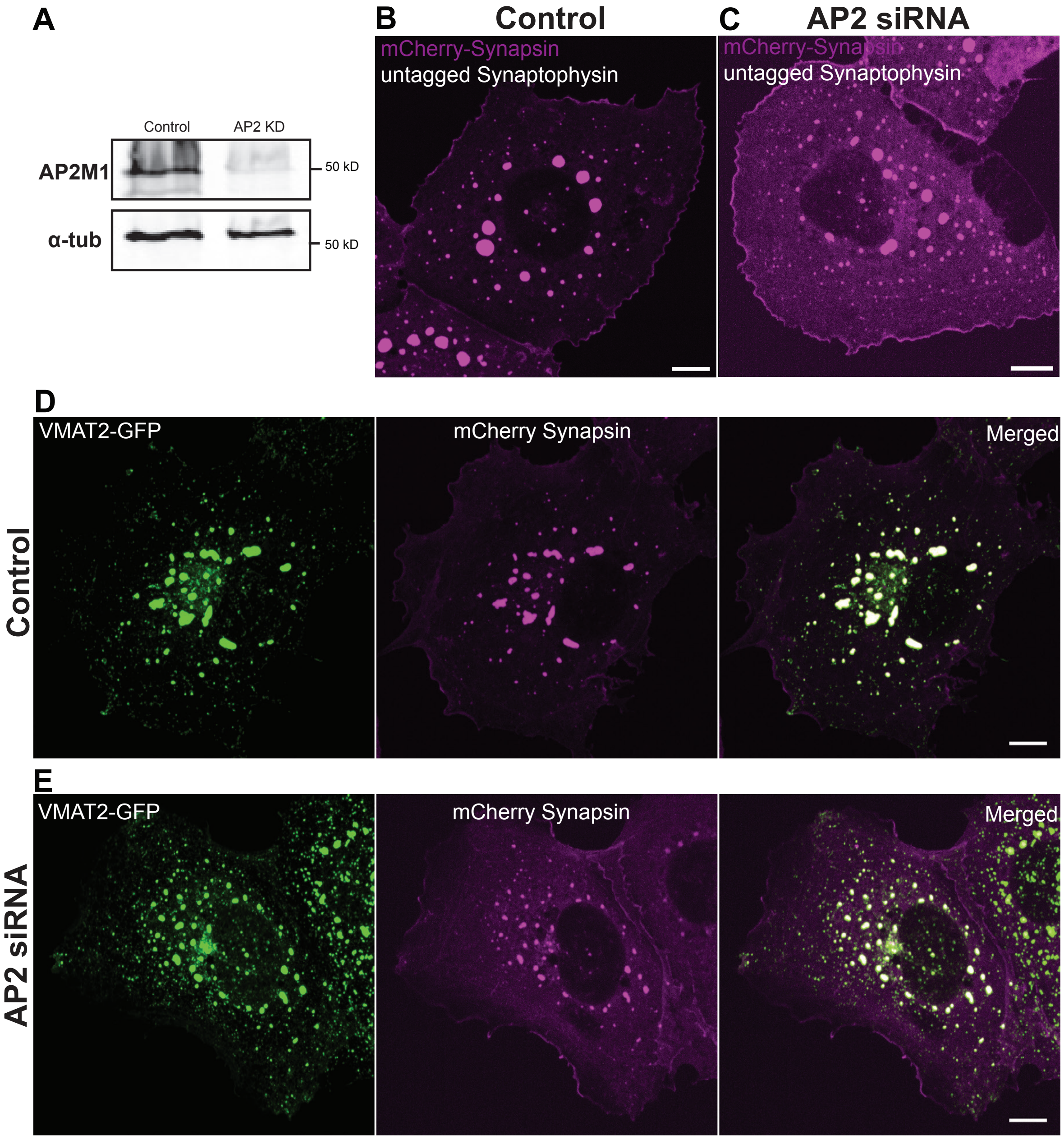

Supplementary Figure 7: AP2 knockdown did not affect assembly of VMAT2-synapsin and synaptophysin-synapsin vesicle clusters

(A) Anti-AP2M1 and α-tubulin (loading control) western blot of control and AP2 KD siRNA-treated COS7 cells 48 hours post-transfection. (B and C) Representative fluorescence images of COS7 cells transfected with untagged synaptophysin and mCherry-synapsin (magenta) treated with control siRNA (B) or AP2M1 siRNA (C). Untagged synaptophysin was identified by the presence of synapsin droplets. (D and E) COS7 cells transfected with VMAT2-GFP (green) and mCherry-synapsin (magenta) treated with control siRNA (D) or AP2M1 siRNA (E). Images from B-E were taken 48 hours after transfection. Scale bars, 10 μm.
